# The Therapeutic Potential of Migrastatin-Core Analogs for the Treatment of Metastatic Cancer

**DOI:** 10.3390/molecules22020198

**Published:** 2017-02-09

**Authors:** Ernest Giralt, Daniele Lo Re

**Affiliations:** 1 Institute for Research in Biomedicine (IRB Barcelona), The Barcelona Institute of Science and Technology, C/Baldiri Reixac 10, Barcelona E-08028, Spain; ernest.giralt@irbbarcelona.org; 2 Department of Organic Chemistry, University of Barcelona, Marti i Franques 1-11, Barcelona E-08028, Spain

**Keywords:** diverted total synthesis, natural products, cancer metastasis

## Abstract

Tumor metastasis is a complex process in which cells detach from the primary tumor and colonize a distant organ. Metastasis is also the main process responsible for cancer-related death. Despite the enormous efforts made to unravel the metastatic process, there is no effective therapy, and patients with metastatic tumors have poor prognosis. In this regard, there is an urgent need for new therapeutic tools for the treatment of this disease. Small molecules with the capacity to reduce cell migration could be used to treat metastasis. Migrastatin-core analogs are naturally inspired macrocycles that inhibit pathological cell migration and are able to reduce metastasis in animal models. Migrastatin analogs can be synthesized from a common advanced intermediate. Herein we present a review of the synthetic approaches that can be used to prepare this key intermediate, together with a review of the biological activity of migrastatin-core analogs and current hypotheses concerning their mechanism of action.

## 1. Introduction

**Figure 1:**
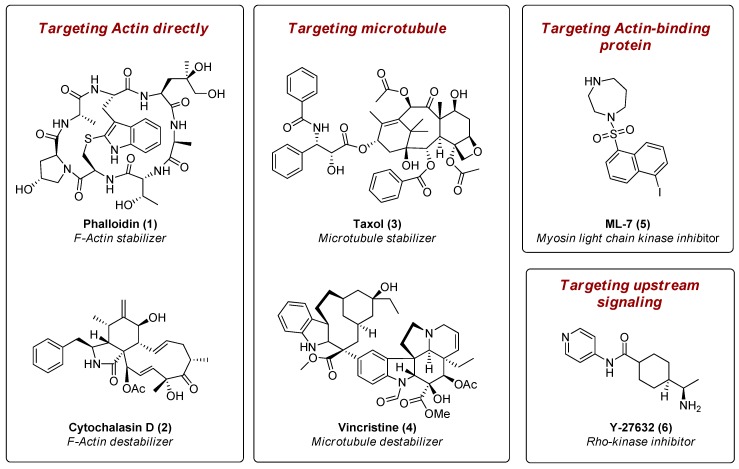
Small-molecule inhibitors of cell mobility.

Cell migration is a physiological process and a central feature in embryonic development, tissue repair and immune cell function. Importantly, cell migration is also responsible for angiogenesis, tumor invasion, and metastasis [1]. The cytoskeleton drives cell migration and a key cytoskeletal component is Actin. Several targets have been proposed for the inhibition of Actin dynamics, including the following: (1) actin (e.g., phalloidin (**1**) an F-actin stabilizer or cytochalasin D (**2**), an F-actin destabilizer); (2) tubulin and microtubule (e.g., taxol (**3**), a microtubule stabilizer or vincristine (**4**), a microtubule destabilizer); (3) actin-binding proteins (ML-7 (**5**), a myosin light chain kinase inhibitor); and (4) upstream signaling molecules (e.g., Y27632 (**6**), a Rho-kinase inhibitor) [2] (Figure 1).

Migrastatin (**7**) is a natural product that was originally isolated from *Streptomyces* sp. MK-929-43F1 [3,4] and later found in fermentation broths of *Streptomyces platensis* [5]. Migrastatin comprises a 14-membered ring macrolactone incorporating two *E* bonds and one *Z* double bond, together with three contiguous stereocenters and a pendant alkyl gluturamide side chain. It has been reported that migrastatin inhibits cell migration [3,4], suppresses multi-drug resistance [6], and antagonizes muscarinic acetylcholine receptor [7].

**Figure 2:**
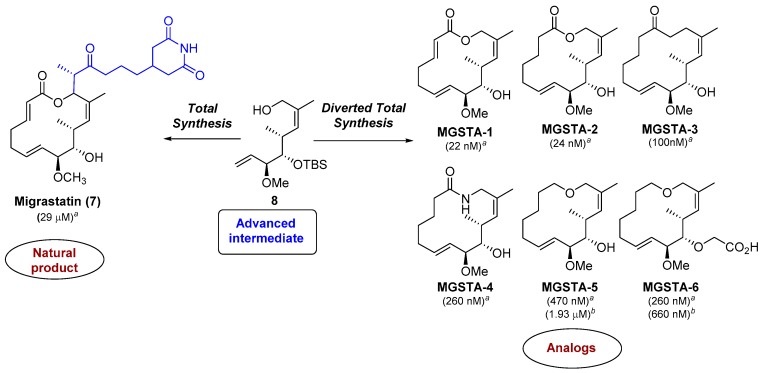
Diverted total synthesis approach for the preparation of migrastatin (**7**) and truncated analogs **MGSTA-1,6** from a common advanced intermediate (**8**). IC_50_ values (in parentheses) in boyden chamber assays against ^a^ 4T1 mouse mammary cancer and ^b^ A549 human lung cancer cells.

In 2003, Danishefsky and co-workers described the first total synthesis of migrastatin [8]. One year later, the same group demonstrated that ablation of the glutaramide side chain increases the biological activity of migrastatin analogs 1000-fold with respect to natural migrastatin [9]. They found that simpler analogs at nanomolar concentrations were able to inhibit cell migration in 4T1 mouse mammary tumor cells. Danishefsky demonstrated that migrastatin and simplified migrastatin analogs could be easily synthesized from a common advanced intermediate **8**. This result highlights the potential of diverted total synthesis drug discovery (Figure 2).

**Figure 3:**
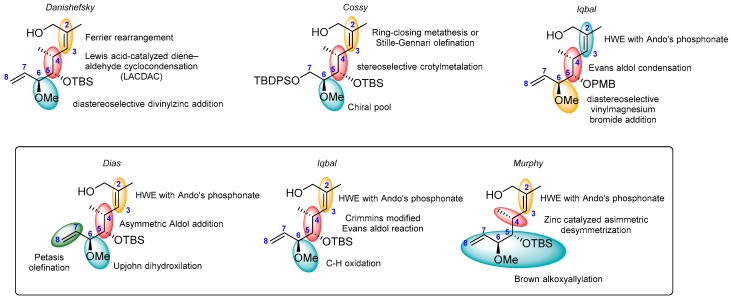
Synthetic strategies for the preparation of advanced intermediate **8**.

Several groups have been involved in the preparation of **8** (Figure 3 and Table 1) [8,9,10,11,12,13,14]. The synthesis and the biological activity of migrastatin (**7**) and migrastatin analogs were covered by Reymond and Cossy in 2008 [15]. The aim of this review is to discuss the recent synthetic approaches used for the preparation of **8**, together with new findings that provide insight into the anti-metastatic potential of migrastatin analogs and their targets.

## 2. Results

### 2.1. Synthesis of Advanced Intermediate ***8***

#### 2.1.1. Synthesis of the Protected Migrastatin-Core (Danishefsky et al.) [16]

**Scheme 1:**
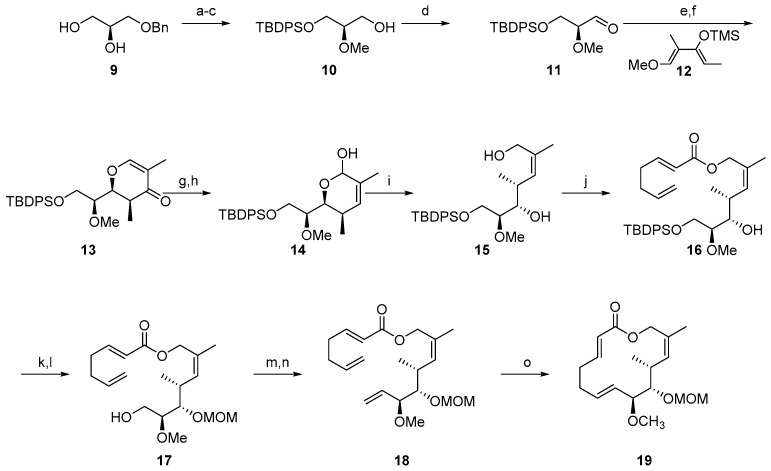
Danishefsky’s synthesis of protected **MGSTA-1** (**19**). *Reagents and conditions*: (**a**) TBDPSCl, imidazole, DMF; (**b**) MeI, NaH, THF; (**c**) H_2_, Pd(OH)_2_, EtOAc, 73% over three steps; (**d**) (COCl)_2_, Et_3_N, DMSO, CH_2_Cl_2_; (**e**) TiCl_4_, CH_2_Cl_2_; (**f**) CSA, PhMe, 71% over three steps; (**g**) NaBH_4_, EtOH, CeCl_3_·7H_2_O; (**h**) CSA, H_2_O, THF; (**i**) LiBH_4_, H_2_O, THF, 55% over three steps; (**j**) (*E*)-hepta-2,6-dienoyl chloride, DMAP, CH_2_Cl_2_, 65%; (**k**) MOMCl, Bu_4_NI, DIPEA, CH_2_Cl_2_; (**l**) HF.py, THF, 79% over two steps; (**m**) DMP, CH_2_Cl_2_; (**n**) Tebbe reagent, pyridine, THF, 60% over two steps; (**o**) Grubbs II catalyst, PhCH_3_, reflux, 50%.

The first synthesis of the migrastatin-core was reported by Danishefsky and co-workers in 2002 [16]. **MGSTA-1** was synthesized as its MOM derivative through a precursor of advanced intermediate **8**. The synthesis began with the selective silylation of the primary hydroxyl group of (*S*)-3-benzyloxy-1,2-propanediol **9** [20,21], followed by methylation of the secondary hydroxyl group. Regioselective deprotection of benzyl ether gave **10**, which was subsequently oxidized under Swern conditions. A Lewis acid-catalyzed diene-aldehyde cyclocondensation (LACDAC) [22] reaction between aldehyde **11** and diene **12** gave dihydropyrone **13**, which contains the three contiguous stereocenters of the migrastatin core fragment. Dihydropyrone **13** was treated with NaBH_4_ and CeCl_3_·7H_2_O (Luche reduction) [23], followed by Ferrier rearrangement in acidic media, to give lactol **14** [24]. Reductive opening of lactol **14** using LiBH_4_ gave compound **15**, which contains the desired (*Z*) olefin. Diol **15** reacted with (*E*)-hepta-2,6-dienoyl chloride in the presence of DMAP to give esther **16**. The secondary hydroxyl group of **16** was protected as MOM ether, and the TBDPS group was cleaved using HF·py to afford intermediate **17**. Oxidation of the primary hydroxyl group using Dess-Martin periodinane (DMP), followed by olefination using the Tebbe reaction [25], gave metathesis precursor **18**. Finally, RCM reaction of **18** with Grubbs II catalyst [26] gave protected migrastatin core compound **19** in 50% yield (Scheme 1).

#### 2.1.2. First Total Synthesis of Migrastatin (Danishefsky et al.) [8]

**Scheme 2:**
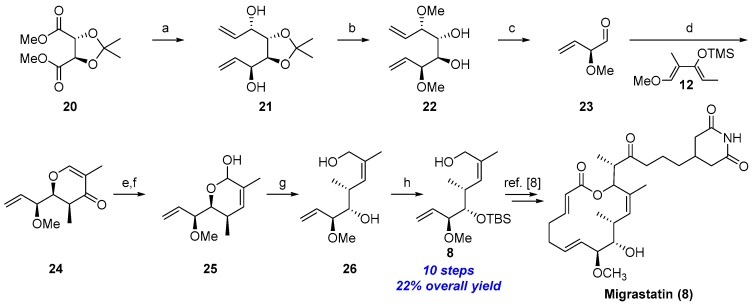
Danishefsky’s synthesis of **8**. *Reagents and conditions*: (**a**) DIBALH, then ZnCl_2_, H_2_C=CHMgBr, PhCH_3_, −78 °C to RT, 75% (ds > 90%); (**b**) (i) MeI, NaH, DMF, RT, (ii) 2 M HCl, MeOH, reflux, 80%; (**c**) Pb(OAc)_4_, Na_2_CO_3_, CH_2_Cl_2_, 0 °C to RT; (**d**) (i) TiCl_4_, CH_2_Cl_2_, −78 °C, (ii) TFA, CH_2_Cl_2_, RT, 87% from **22**; (**e**) LiBH_4_, MeOH, THF, −10 °C; (**f**) CSA, H_2_O, THF, reflux; (**g**) LiBH_4_, H_2_O, THF, RT, 53% from **24**; (**h**) TBSOTf, 2,6-lutidine, CH_2_Cl_2_, RT, (ii) AcOH:H_2_O:THF (3:1:1), RT, 80%.

In 2003, Danishefsky and co-workers publish the first total synthesis of migrastatin (**7**) [8]. In this regard, migrastatin was obtained from advanced intermediate **8**, which was prepared using a similar strategy as that used for the synthesis of **15**. A slightly optimized procedure for the preparation of **8** was reported one year later [9] and is illustrated in Scheme 2.

The synthesis [9] began with commercially available dimethyl 2,3-*O*-isopropylidene-l-tartrate (**20**), which was reduced using DIBAL. Diastereoselective divinylzinc addition afforded the desired vinyl carbinol **21** [27]. The two free hydroxyl groups were then methylated and the acetonide protecting group was removed to afford diol **22** [28]. Glycol cleavage using Pb(OAc)_4_ led to vinyl aldehyde **23**, which was then used for the LACDAC sequence. Aldehyde **23** was reacted with diene **12** in the presence of TiCl_4,_ affording dihydropyrone **24** as a single diastereoisomer [22]. As above, the reduction of **24**, followed by Ferrier rearrangement [24], gave lactol **26**, which, after reductive opening using LiBH_4_ and protection of secondary alcohol as TBS ether, afforded advanced intermediate **8**. With **8** in hand, migrastatin was finally synthesized (Scheme 2). The synthetic procedure leading to migrastatin has already been reviewed elsewhere [15].

#### 2.1.3. Synthesis of the Migrastatin-Core Library (Danishefsky et al.) [9,29]

**Scheme 3:**
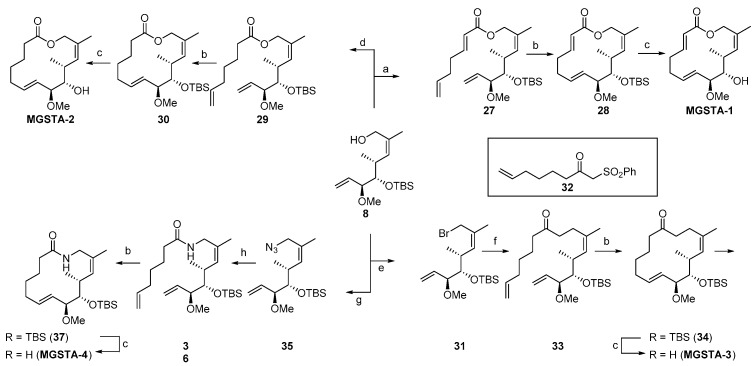
Danishefsky’s synthesis of the migrastatin-core library. *Reagents and conditions*: (**a**) (*E*)-hepta-2,6-dienoic acid, 2,4,6-trichlorobenzoyl chloride, DIPEA, pyridine, PhCH_3_, RT, 48%; (**b**) Grubbs II catalyst (20 mol %), PhCH_3_ (0.5 mM), reflux, 55% (**28**), 76% (**30**), 81% (**34**), 60% (**37**); (**c**) HF^.^pyridine, THF, RT, 66% (**MGSTA-1**), 94% (**MGSTA-2**), 90% (**MGSTA-3**), 81% (**MGSTA-4**); (**d**) 6-heptenoyl chloride, DMAP, CH_2_Cl_2_, RT, 82%; (**e**) CBr_4_, solid-supported PPh_3_, CH_2_Cl_2_, RT; (**f**) (i) β-ketosulfone **32**, DBU, PhCH_3_, RT, (ii) Na/Hg, Na_2_HPO_4_, MeOH, RT, 61% from **7**; (**g**) DPPA, DBU, PhCH_3_, RT, 87%; (**h**) (i) PPh_3_, H_2_O, THF, 70 °C, (ii) 6-heptenoic acid, EDC, DIPEA, CH_2_Cl_2_, RT, 92%.

After achieving the total synthesis of migrastatin [8], Danishefsky and co-workers reported [9,29] the preparation of a small library of migrastatin-core analogs using advanced intermediate **8** (Scheme 3). Reaction of **8** with (*E*)-hepta-2,6-dienoic acid in the presence of 2,4,6-trichlorobenzoyl chloride and DIPEA gave acylated compound **27**, which, after RCM and protecting group removal, afforded **MGSTA-1**.

A similar strategy was applied for the preparation of **MGSTA-2**. Intermediate **8** was coupled to 6-heptenoyl chloride to give **29**, which, after RCM and de-protection, afforded **MGSTA-2**. For the preparation of macroketone analogs, intermediate **8** was converted into allylic bromide **31** using the Appel reaction. Compound **31** was then coupled to β-ketosulfone **32** [30], followed reductive removal of the sulfone group mediated by Na/Hg, to give ketone **33**. RCM and de-protection of **33** gave **MGSTA-3**. For the preparation of macrolactam analogs, compound **8** was converted into allylic azide **35** using diphenylphosphoryl azide under Mitsunobu conditions. Staudinger reduction of **35**, followed by coupling with 6-heptenoic acid in the presence of EDC, gave compound **36,** which, after RCM and removal of silyl protecting group, afforded **MGSTA-4** (Scheme 3).

#### 2.1.4. Synthesis by Cossy et al. [10,17]

**Scheme 4:**
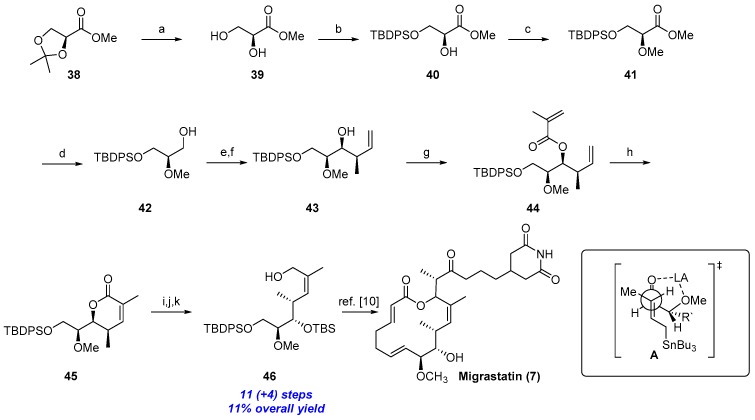
Cossy’s synthesis of **46**. *Reagents and conditions*: (**a**) *p*TSA, MeOH/H_2_O (1:1), RT, 83%; (**b**) TBDPSCl, imidazole, CH_2_Cl_2_, 0 °C to RT 81%; (**c**) Ag_2_O, MeI, MS 4 Å, Et_2_O, 40 °C, 96%; (**d**) DIBAL, CH_2_Cl_2_, −78 °C to RT 90%; (**e**) (COCl)_2_, DMSO, CH_2_Cl_2_, −78 °C, then Et_3_N −78 °C to RT; (**f**) MgBr_2_**·**OEt_2_, CH_2_Cl_2_, −20 °C, then but-2-enyl-[(tri(*n*-butyl)]stannane, −60 °C, 87% (over two steps); (**g**) methacryloyl chloride, Et_3_N, DMAP, CH_2_Cl_2_, 0 °C to RT 80%; (**h**) Grubbs II catalyst (16.5 mol %), CH_2_Cl_2_ (*c* = 10^−2^ M), 40 °C, 144 h, 65%; (**i**) LiBH_4_ (7 equiv.), CeCl_3_·7H_2_O (1 equiv.), THF/H_2_O (4:1), RT, 74%; (**j**) TBSOTf, 2,6-lutidine, CH_2_Cl_2_, 0 °C to RT, 75%; (**k**) THF/H_2_O/AcOH (1:1:3), 36 h, RT, 75%.

In 2006, Cossy and co-workers [10] reported the synthesis of a precursor **46** of advanced intermediate **8** (Scheme 4). The synthesis began from methyl esther **38**. Acetonide cleavage of **38** followed by regioselective protection of primary alcohol in **39** as TBDPS ether afforded **40**. Methylation of secondary alcohol in **40**, followed by DIBAL reduction of the ester moiety, gave primary alcohol **42**. After oxidation of **42** under Swern conditions, the corresponding aldehyde was treated with 2-enyl[tri(*n*-butyl)]stannane the presence of MgBr_2_·OEt_2_ to give the *syn*,*syn*-stereotriad **43** with good stereocontrol (*dr* = 90:10) [31]. The authors claim that the stereochemical outcome of that reaction resulted from an open chair transition state of type **A**, where the carbonyl and the methoxy group of aldehyde are chelated with MgBr_2_·OEt_2_ [31]. Compound **43** was treated with methacryloyl chloride in the presence of TEA and DMAP to give diene **44**. RCM of **44** using Grubbs II catalyst gave unsaturated lactone **45**, which contains the olefin with desired *Z* configuration and three contiguous stereocenters. Treatment of **45** with LiBH_4_ in the presence of CeCl_3_·7H_2_O, followed by simultaneous protection of the two hydroxyl groups as TBS ether and regioselective de-protection of the primary alcohol, afforded precursor **46**. This compound was therefore converted into migrastatin **7** using a similar procedure to that described by Danishefsky and co-workers [8] (Scheme 4).

**Scheme 5:**
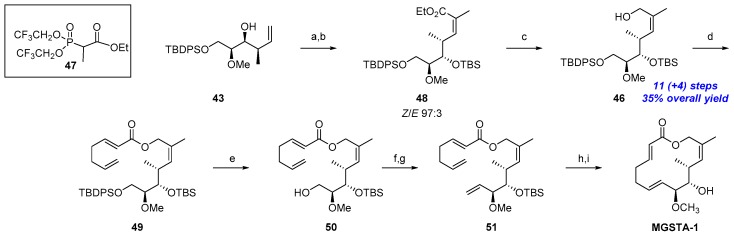
Synthesis of **MGSTA-1**. *Reagents and conditions*: (**a**) TBSOTf, 2,6-lutidine, CH_2_Cl_2_, −20 °C, 93%; (**b**) (i) OsO_4_, NMO, *t*-BuOH/H_2_O (1/1), RT; (ii) NaIO_4_, THF/H_2_O (1/1), RT; (iii) **47**, KHMDS, 18-crown-6, THF, −78 °C, 80% (over 3 steps); (**c**) DIBAL, CH_2_Cl_2_, −78 °C to RT, 94%; (**d**) (*E*)-2,4,6-trichlorobenzoic (*E)*-hepta-2,6-dienoic anhydride, Pyridine, PhCH_3_, RT, 67%; (**e**) NH_4_F MeOH, reflux, 77%; (**f**) Dess-Martin Periodinane CH_2_Cl_2_, 0 °C to RT; (**g**) Zn, PbCl_2_ cat, CH_2_I_2_Ti(O*i*-Pr)_4_, THF, RT (**h**) Grubbs II catalyst (20 mol %), PhCH_3_, reflux, 47%; (**i**) HF Py, THF, RT, 67%.

In 2007, Cossy and co-workers [17] reported the synthesis of **46** using a Still–Gennari olefination to control the formation of the (*Z*)-double bond (Scheme 5). Compound **43** [10] was protected as TBS ether and therefore subjected to oxidative cleavage using OsO_4_, followed by treatment with NaIO_4_. The aldehyde obtained was treated with Still-Gennari phosphonate [32,33] in the presence of KHMDS to give the unsaturated ester **48** with a good *Z*/*E* ratio (97:3). Reduction of **48** with DIBAL gave the precursor **46**. Following similar steps to those reported by Danishefsky et al. [9], this compound was converted to **MGSTA-1** in 5 steps (Scheme 5).

#### 2.1.5. Synthesis by Iqbal et al. [11]

In 2006, Iqbal and Parthasarati reported the synthesis of advanced intermediate **8** as its PMB derivative **65** in 12 steps. The synthesis started with dibutylboron triflate-mediated Evans aldol condensation of acrolein **52** and (*S*)-benzyl oxazolidinone **53** to give the desired aldol product **54** [34].

**Scheme 6:**
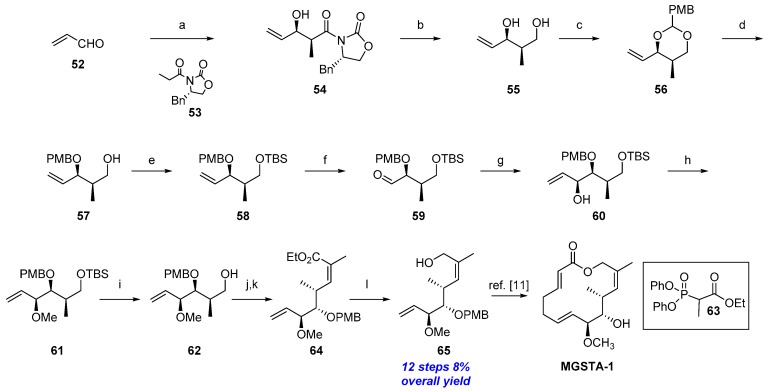
Iqbal’s synthesis of **65**. *Reagents and conditions*: (**a**) *n*-Bu_2_BOTf, *i*-Pr_2_NEt, CH_2_Cl_2_, −78 °C to 0 °C, 1 h, 84%; (**b**) LiBH_4_, MeOH, THF, 0 °C, 2 h, 96%; (**c**) (OMe)_2_CHC_6_H_4_OMe, CSA, CH_2_Cl_2_, RT, 12 h, 70%; (**d**) DIBAL, CH_2_Cl_2_, −78 °C to 0 °C, 2 h, 95%; (**e**) TBSCl, imidazole, DMF, RT, 12 h, 88%; (**f**) OsO_4_, NaIO_4_, 2,6-lutidine, dioxane/H_2_O (3:1), RT, 3 h, 82%; (**g**) H_2_C=CHMgBr, MgBr_2_·OEt_2_, CH_2_Cl_2_, RT, 2 h, 72%; (**h**) MeOTf, 2,6-di-*tert*-butyl-4-methylpyridine, CH_2_Cl_2_, reflux, 6 h, 62%; (**i**) TBAF, THF, RT, 12 h, 94%; (**j**) DMP, CH_2_Cl_2_, RT, 40 min, 85%; (**k**) (PhO)_2_P(O)CH(CH_3_)CO_2_C_2_H_5_, DBU/NaI, THF, −78 °C to 0 °C, 3 h, 60%; (**l**) DIBAL, CH_2_Cl_2_, −78 °C, 1 h, 96%.

The chiral auxiliary was then removed using LiBH_4_ in THF to yield diol **55,** which was then protected with 4-methoxybenzaldehyde dimethyl acetal [35] to afford compound **56**. Acetal **56** was opened using DIBAL [35], and the corresponding primary alcohol **57** was then protected as TBS ether to afford the orthogonally protected compound **58**. Oxidative cleavage of **58** using OsO_4_-NaIO_4_ in the presence of 2,6-lutidine [36] gave aldehyde **59** in good yield. Lewis acid-mediated distereoselective addition of vinylmagnesium bromide to aldehyde **59** gave the desired compound **60** (*dr* = 7:1), which was separated from its diastereomer in the next step. Secondary alcohol **60** was methylated using MeOTf [37] to yield enantiomerically pure **61**. TBAF-mediated deprotection of the TBS group in **61,** followed by oxidation using Dees-Martin periodinane [38], gave the corresponding aldehyde, which was treated with Ando’s phosphonate **63** [39] in the presence of DBU to afford the (Z)-olefin **64** (*Z/E* ratio not reported). Reduction of the ester moiety using DIBAL gave the advanced intermediate **65,** which was used for the preparation of the migrastatin core **MGSTA-1** (Scheme 6).

#### 2.1.6. Synthesis by Dias et al. [12]

**Scheme 7:**
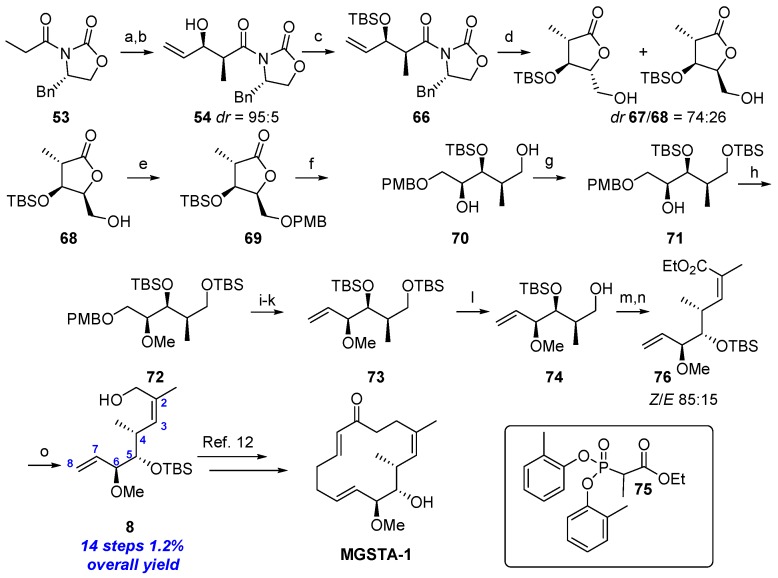
Dias’ synthesis. Synthesis of **8**. *Reagents and conditions*: (**a**) TiCl_4_, DIPEA, CH_2_Cl_2_, −78 °C to 0 °C; 1 h; (**b**) acrolein, −78 °C to 0 °C to RT, 12 h, 87% over two steps; (**c**) TBSOTf, 2,6-ludine, CH_2_Cl_2,_ 0 °C, 20 min, 93%; (**d**) NMO, OsO4, cat., acetone/H_2_O, 0 °C, 45 min, **67** (57%), **68** (20%); (**e**) CSA cat., 4-methoxybenzyl-2,2,2-trichloroacetimidate, CH_2_Cl_2_, RT, 12 h, 67%; (**f**) LiAlH_4_, THF, −78 °C, 1 h, 75%; (**g**) TBSCl, imidazole, CH_2_Cl_2_, 0 °C, 1 h, 95%; (**h**) proton sponge, Me_3_OBF_4_, CH_2_Cl_2_, RT, 12 h, 75%; (**i**) DDQ/H_2_O, CH_2_Cl_2_; RT, 2 h, 88%; (**j**) NMO, TPAP cat., CH_2_Cl_2_; RT, 1 h; (**k**) Cp_2_TiMe_2_, PhCH_3_, 70 °C, 12 h, 50% over two steps; (**l**) HF-Py-THF, THF, RT, 12 h; 80%; (**m**) NMO, TPAP cat., CH_2_Cl_2_; 1 h, RT, (**n**) **75** in THF, NaH, RT, 12 h, 58% over two steps; (**o**) DIBAL, CH_2_Cl_2_, −15 °C, 1 h, 98%.

The strategy used by Dias and co-workers for the preparation of advanced intermediate **8** involved the formation of stereocenters at C4,5 by means of an asymmetric aldol addition. The methoxy group at C6 was introduced as hydroxyl group using Upjohon dihydroxylation and, finally, *Z* configuration at C2,3 was introduced using a HWE reaction. The synthesis began with asymmetric aldol addition of a titanium enolate derived from (*S*)-4-benzyl-3-propionyloxazolidin-2-one **53** which was reacted with acrolein **52** to give product **54** in 87% (*dr* = 95:5) [40,41,42,43,44,45,46,47,48]. The secondary alcohol was then protected as TBS ether to afford **66** in 93% yield. Treatment of **66** with 4-methylmorpholine oxide and a catalytic amount of OsO_4_ in acetone/H_2_O at 0 °C [49] afforded lactones **67** and **68** (*dr* = 74:26) on a multigram scale (Scheme 7).

After chromatographic separation and recovery of the chiral auxiliary, lactone **68** was reacted with 4-methoxybenzyl 2,2,2 trichloroacetimidate in the presence of 10-camphorsulfonic acid to afford fully protected lactone **69**. Reduction of **69** with LiAlH_4_ gave primary alcohol **70** in good yield. The primary alcohol in **70** was then protected as TBS ether (**71**), and the free secondary alcohol was methylated using 1,8-bis(dimethylamino)naphthalene (proton sponge) [50] to afford compound **72**. The *p*-methoxybenzyl protecting group was then selectively removed using DDQ/H_2_O. Ley’s oxidation of the resulting primary alcohol [51] followed by Petasis olefination [52] gave olefin **73** in 44% yield (from **72**). The primary TBS group was regioselectively removed using HF·Py·THF to afford the corresponding primary alcohol **74**. Ley’s oxidation followed by HWE reaction using Ando’s phosphonate ester **75** [39,53,54] gave compound **76** (*Z*/*E* = 85:15) in 58% yield (~100 mg scale) from **74**. Finally, reduction of the ester moiety using DIBAL afforded the advanced intermediate **8** in 14 steps with 1.2% overall yield. Using **8**, Andricopulo et al. prepared the macrolactone of migrastatin, namely **MGSTA-1**. The coupling of **8** to (*E*)-2,6-heptadienoic acid using DCC and DMAP [55,56,57], followed by ring-closing metathesis (RCM) [8,9,29] and removal of TBS protecting group, afforded **MGSTA-1**.

**Scheme 8:**
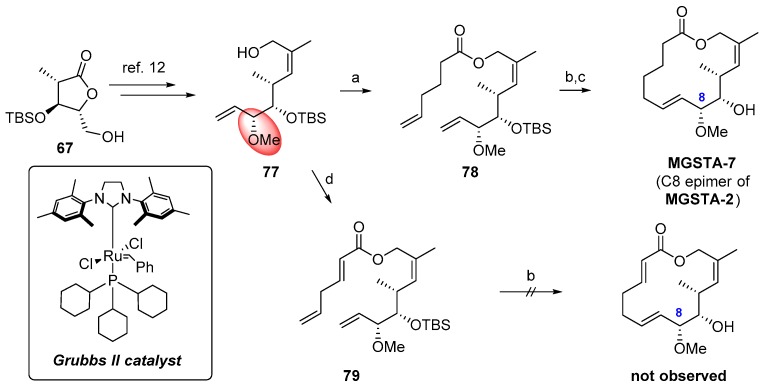
Synthesis of C8 epimer of **MGSTA-2**. *Reagents and conditions*: (**a**) DCC, DMAP, 6-heptanoic acid, CH_2_Cl_2_, RT, 12 h, 92%; (**b**) Grubbs II catalyst, PhCH_3_, reflux, 30 min, 80%; (**c**) HF, CH_2_Cl_2_/CH_3_CN, RT, 24 h, 40%; (**d**) DCC, DMAP, (*E*)-2,6-heptadienoic acid, CH_2_Cl_2_, RT, 98%.

Since undesired lactone **67** was prepared on multi-gram scale, the authors undertook the preparation of the C-8 epimer of the migrastatin core, **MGSTA-7**. Lactone **67** was converted to compound **77** using the same strategy as that described above for the preparation of **8**. Coupling of **77** to 6-heptadienoic acid gave **78,** which underwent RCM. After removal of the TBS protecting group, **78** afforded **MGSTA-7**. Interestingly, **77** was coupled to (*E*)-2,6-heptadienoic acid, and the resulting compound **79** was submitted to RCM using Grubbs II catalyst . However, no metathesis product was observed (Scheme 8).

#### 2.1.7. Synthesis by Iqbal et al. [13]

**Scheme 9:**
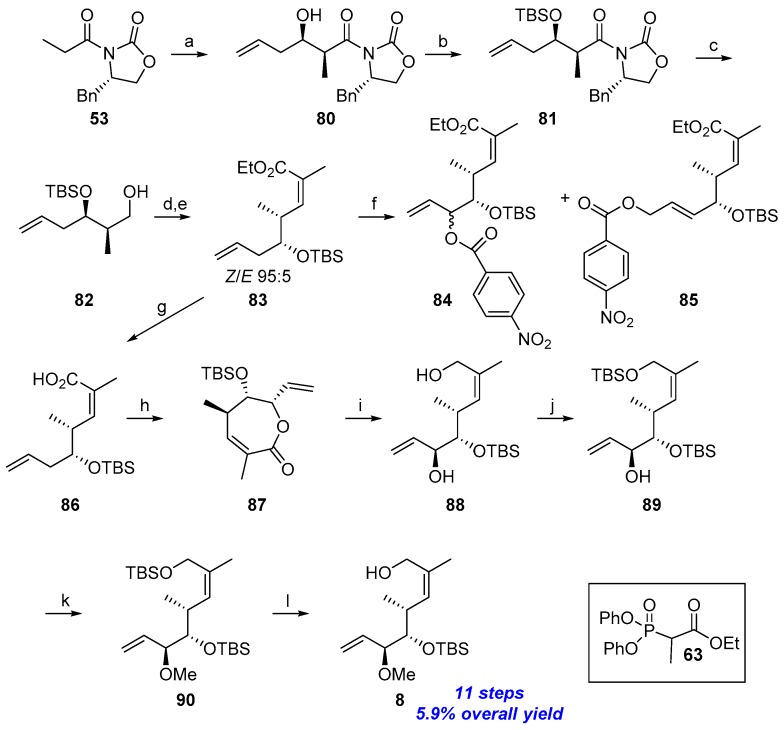
Iqbal’s synthesis of **8**. *Reagents and conditions*: (**a**) TiCl_4_, 3-butenal, (−) spartein, CH_2_Cl_2_, 0 °C, 1 h, 83%; (*dr* 20:1); (**b**) TBSOTf, DIPEA, CH_2_Cl_2_, 0 °C, 90%; (**c**) NaBH_4_, THF:H_2_O, 90 h, 80%; (**d**) DMP, CH_2_Cl_2_, 2 h, 90%; (**e**) **63**, NaI, DBU, −78 °C to 0 °C, THF, 3 h, *Z:E*, 95:5, 60%; (**f**) White catalyst 10%, *p*-benzoquinone, *p*-nitrobenzoic acid, 45 °C, 72 h, **84** (24%) and **85** (20%); (**g**) LiOH, THF/MeOH/H_2_O, 4 h, 55 °C, 90%; (**h**) White catalyst 10%, *p*-benzoquinone, Cr(III)salenCl, 1,4-dioxane, 45 °C, 72 h, 40% (78% based on recovered starting material); (**i**) DIBAL, −78 °C to 0 °C, CH_2_Cl_2_, 2 h, 90%; (**j**) TBSCl, Imidazole, DMF, 2 h, 90%; (**k**) DTBMP, CH_2_Cl_2_, MeOTf, 6 h, reflux, 70%; (**l**) CSA, MeOH 2 h, 90%.

Iqbal’s synthesis of advanced intermediate **8** began with an Crimmins modified Evans aldol reaction [43,58] of Evan’s chiral auxiliary **53** and 3-butenal in the presence of (−)-spartein and TiCl_4_ to give aldol adduct **80**. The secondary alcohol was protected as TBS ether (**81**), and the chiral auxiliary was removed using sodium borohydride to afford primary alcohol **82**. Hence, oxidation of **82** using Dess-Martin periodinane gave the corresponding aldehyde, which reacted with ethyl 2-(diphenyl-phosphono)acetate (**63**) [59] in the presence of DBU to give the corresponding α,β-unsaturated ester **83** with *Z* configuration. The attempt to promote intermolecular allylic C-H oxidation on ester **83** using White’s catalyst [60] gave two regioisomers **84** and **85** in equal ratio [61]. Compound **84** was characterized and appeared as a single product (^1^H-NMR evidence); however, no further studies were performed to assign the stereochemistry of the newly formed stereocenter. With the aim to achieve high regio- and diastereo-selectivity, the authors hydrolyzed ester **83** to the corresponding carboxylic acid **86,** which was then submitted to intramolecular lactonization via C-H allylic activation using White’s catalyst [62,63,64]. After a brief optimization, the authors found that the reaction of **86** with White’s catalyst in the presence of DDQ and Cr(III)salenCl gave lactone **87** as a single diastereoisomer in 40% yield with 50% recovery of the starting material. Reduction of lactone **87** with DIBAL gave the corresponding diallylic alcohol **88**. Regioselective protection of primary alcohol was achieved using TBSCl in the presence of imidazole to afford **89**. Methylation of the secondary alcohol using MeOTf gave intermediate **90**. Finally, regioselective deprotection of primary alcohol with CSA in methanol gave advanced intermediate **8** (Scheme 9).

#### 2.1.8. Synthesis by Murphy et al. [14]

**Scheme 10:**
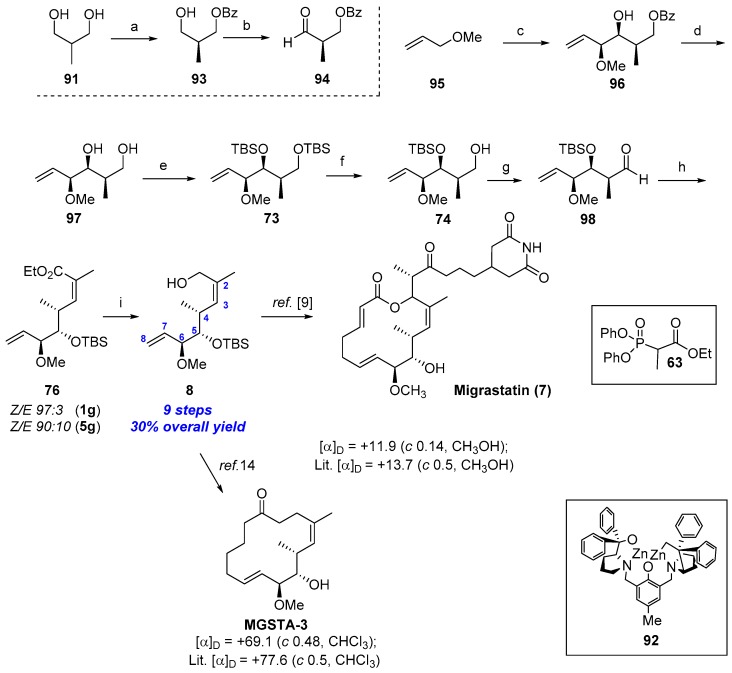
Murphy’s synthesis of **8**. *Reagents and conditions*: synthesis of **8**, migrastatin **7** and **MGSTA-3**. Reagents and conditions: (**a**) vinyl benzoate, **92**, PhCH_3_, −20 °C, 48 h, 89%; (**b**) DAIB, TEMPO, CH_2_Cl_2_, 2.5 h, RT, 84%; (**c**) (i) *s*BuLi, THF, 15 min, −78 °C; (ii) (+)Ipc_2_BOMe, −78 °C, 1 h; (iii) BF_3_·Et_2_O then **94**, −78 °C, 20 h, then 1 M NaOH, 30% H_2_O_2_, RT, 20 h; (**d**) K_2_CO_3_, MeOH, RT, 18 h, 73% from **94**; (**e**) TBSOTf, Et_3_N, CH_2_Cl_2_, 0 °C, 1.5 h; (**f**) *p*-TSA, MeOH, 0 °C, 2 h, 84% over two steps; (**g**) Dess–Martin reagent, CH_2_Cl_2_, pyridine, RT, 18 h, 93%; (**h**) **63** in THF, NaH, 0 °C, 1.5 h, then **98** at −78 °C, 30 min, then 0 °C for 15 h, 79%; (**i**) DIBAL, CH_2_Cl_2_, −78 °C, 10 min, 90%.

In their synthesis of fragment **8**, Murphy and co-worker used Ando’s phosphonate ester [11,12,13,39,59] to introduce the C2,3 olefin with *Z* configuration. A diastereoselective Brown alkoxylallylation [65] was used to introduce the two contiguous stereocenters at C5,6. The synthesis began with desymmetrization of 2-methylpropanediol **91** using a scale-up of the method reported by Trost et al. [66] Diol **91** was treated with *S*,*S*-Cat **92** and vinyl benzoate to afford monoprotected alcohol **93**. Subsequent oxidation of **93** using TEMPO and BAIB gave aldehyde **94**. Hence, allyl methyl ether was treated with *s*BuLi at low temperature. Addition of (+)-*B*-methoxydiisopino-campheylborane gave the corresponding borane, which reacted with chiral aldehyde **94** to yield **96** with high diastereoselectivity. The benzoate protecting group was then removed [67] to give diol **97** as a single diastereoisomer in good yield. Both hydroxyl groups were protected as TBS ethers using TBSOTf in the presence of triethylamine (**73**). Regioselective de-protection of the primary TBS protecting group was achieved by treating the fully protected **73** with catalytic *p*-toluenesulfonic acid in methanol to give **74**. Oxidation of **74** using Dess-Martin periodinane afforded aldehyde **98** in excellent yield. Horner-Wadsworth-Emmons reaction was used to introduce the Z olefin at C2,3. Treatment of **98** with Ando’s phosphonate **63 [11,13]** gave the desired alkene **76**. The reaction proceeded with high *Z* selectivity (*Z*/*E* = 97:3) on a 1.0 g scale. The authors reported some erosion of the Z selectivity when the reaction was scaled up to ~4.0 g (*Z*/*E* = 90:10). Finally, reduction of ester moiety using with diisobutylaluminum hydride (DIBAL-H) afforded intermediate **8** in 9 steps with an overall yield of 30% from **91**. Using **8**, the authors prepared Macroketone **MGSTA-3** in 100-mg scale, which was used for in vivo studies [14], and the natural product migrastatin (**7**) (Scheme 10). Interestingly, **MGSTA-3**, which was contaminated by hydrocarbon and silicon grease [68], was purified by distillation under reduced pressure using a Kugelrohr apparatus (0.05 mbar, T = 90 °C, 18 h).

In 2015 [18], Murphy and co-workers reported the preparation of several migrastatin-core analogs with variations in the macrocycle ring size and functionality. Therefore, advanced intermediate **8** was used for the preparation of compounds **MGSTA-8** to **MGSTA-16**.

**Scheme 11:**
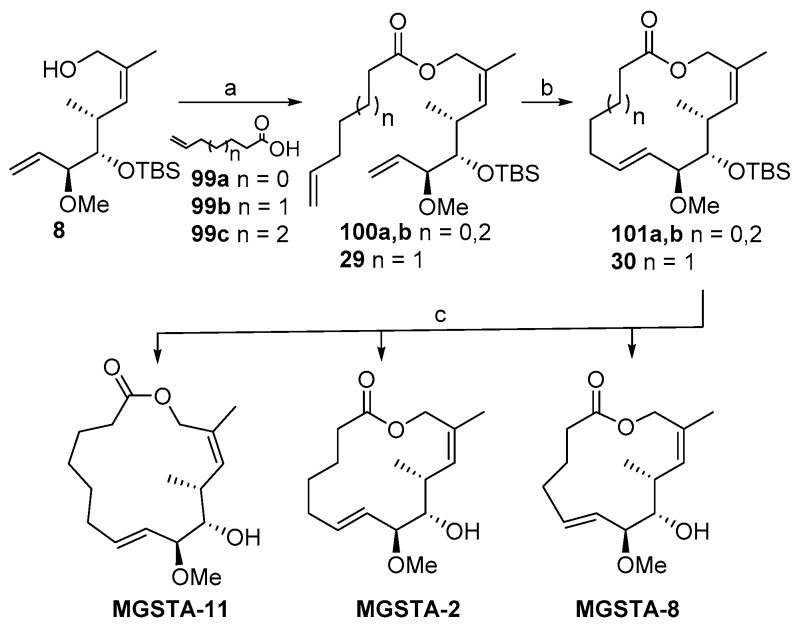
Synthesis of macrolacton migrastatin-core analogs. *Reagents and conditions*: (**a**) **99a**–**c**, Ph_3_P, DIAD, PhCH_3_, RT; **100a** (69%), **100b** (76%); (**b**) Grubbs II catalyst, PhCH_3_, reflux, **101a** (68%), **101b** (73%); (**c**) HF.py, THF, RT; **MGSTA-8** (61%), **MGSTA-11** (54%).

The coupling of advanced intermediate **8** with carboxylic acids **99a**–**c** under Mitsunobu conditions gave the corresponding esters **29** and **100a,b** which underwent ring-closing metathesis (RCM) using Grubbs II catalyst to give macrolactones **30** and **101a,b**. Removal of the TBS group was achieved using HF·pyridine to afford **MGSTA-2** and the 13- and 15-membered analogs **MGSTA-8** and **MGSTA-11** (Scheme 11).

**Scheme 12:**
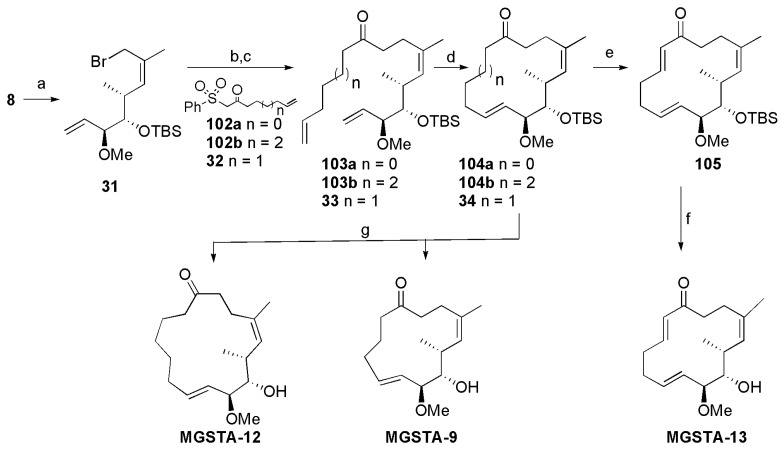
Synthesis of macroketone migrastatin-core analogs. *Reagents and conditions*: (**a**) CBr_4_, Ph_3_P polymer-bound, CH_2_Cl_2_; (**b**) **32-102a,b**, DBU, PhCH_3_ then **31**, RT; (**c**) Na/Hg, MeOH, RT; (**103a** (51%) **103b** (51%), from **8**; (**d**) Grubbs II catalyst, PhCH_3_, reflux, **104a** (99%), **104b** (60%); (**e**) (i) **34**, TMSCl, LHMDS, THF, 0 °C, 2 h; (ii) Pd(OAc)_2_, CH_3_CN, RT, 2 h, 78% from **34**; (**f**) HF·Py, THF, RT, **MGSTA-13** (90%); (**g**) HF·Py, THF, RT, **MGSTA-9** (84%), **MGSTA-12** (82%).

Furthermore, migrastatin-core-based macroketones were prepared by the conversion of allylic alcohol **8** into the allylic bromide **31** using an Appel reaction [9]. This was subsequently reacted with β-ketosulfones **32**-**102a,b [9]** to give ketones **33** and **103a,b**. Ring-closing metathesis reaction using Grubbs II catalyst afforded macroketones **34** and **104a,b,** which, after TBS removal using HF·Py, gave **MGSTA-3**, and the 13- and the 15-membered analogs **MGSTA-9** and **MGSTA-12**. In addition, protected macroketone **34** underwent Saeugusa Ito oxidation using LHMDS and TMSCl, followed by treatment with Pd(OAc)_2_, to give the α,β-unsaturated macroketone **MGSTA-13** after TBS de-protection (Scheme 12).

**Scheme 13:**
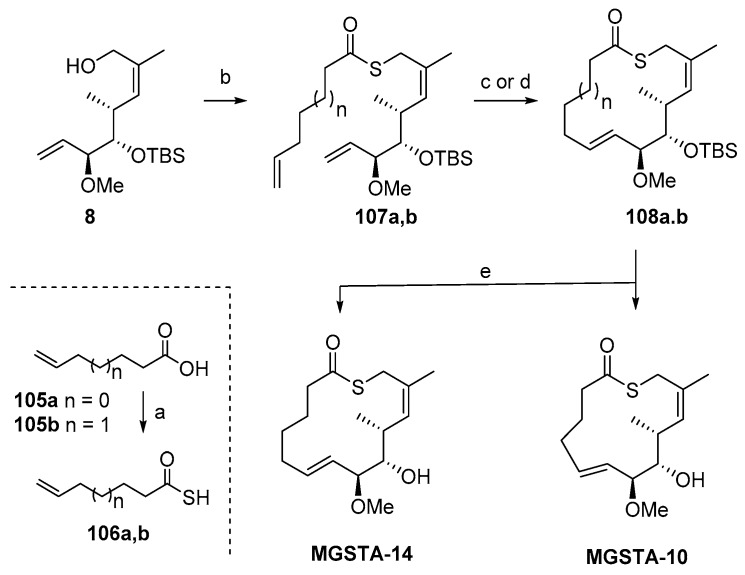
Synthesis of macrothiolactone migrastatin-core analogs. *Reagents and conditions*: (**a**) Lawesson’s reagent, CH_2_Cl_2_, mw, 100 °C, 10 min; (**b**) **105a,b**, Ph_3_P, DIAD, PhCH_3_, RT; **106a** (42%), **106b** (39%); (**c**) Grubbs II catalyst, CH_2_Cl_2_, mw, 100 °C, 30 min, **107b** (49%); (**d**) Grubbs-II catalyst, PhCH_3_, reflux, **107a** (88%); (**e**) HF·Py, THF, RT; **MGSTA-3** (85%), **MGSTA-7** (63%).

Macrothiolactones were also prepared using Mitsunobu reaction of thioacids **106a,b** with allylic alcohol **8**. Thioacids **106a** and **106b** were prepared by treating the corresponding carboxylic acids **105a,b** with Lawesson’s reagent under microwave irradiation [69]. As described above, RCM and removal of the TBS group afforded macrothiolactones **MGSTA-10** and **MGSTA-14** (Scheme 13). Interestingly, silicon grease impurity in **MGSTA-8** to **MGSTA-14** was removed after distillation under reduced pressure (Kugelrohr apparatus: 0.05 mbar, T = 90 °C, 2 to 24 h). Since **MGSTA-3** was synthesized in 100-mg scale, the authors undertook the preparation of glucuronidated migrastatin-core analogs.

**Scheme 14:**
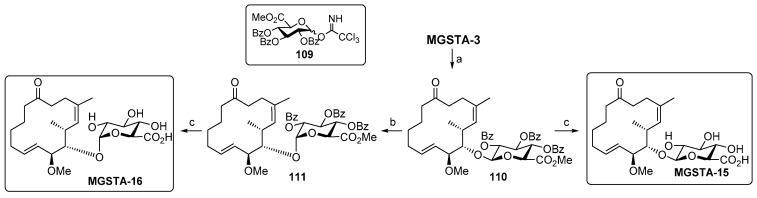
Reagents and conditions: (**a**) **109** TMSOTf, MS (AW300), CH_2_Cl_2_, −78 °C, 5 h, 60%; (**b**) TiCl_4_, CDCl_3_, 4 °C, 69%; (**c**) NaOH aq., MeOH, RT, 18 h, **MGSTA-16** (61%), **MGSTA-17** (73%).

The glucuronidation of small molecules is a physiological process that leads to highly water-soluble compounds that are therefore excreted through the kidney [70]. These compounds are generally biologically inactive. However, it has been found that glucuronidated small molecules can display increased biological activity through a direct or indirect mechanism [70,71,72]. In addition, β-glucuronidases are generally overexpressed in tumor tissue and glucuronidation strategy has been exploited for the preparation of pro-drugs based on SN-38 or taxol [73,74]. Murphy and co-workers demonstrated that reaction of **MGSTA-3** with trichloroacetimidate **109** [75] in the presence of TMSFOTf leads to the protected β-glucuronide **110**. The resulting compound was subsequently treated with TiCl_4_ [76,77,78,79,80,81,82] to give α-glucuronide **111** as a single product. Finally, removal of benzoate groups and hydrolysis of methyl ester led to migrastatin analogs **MGSTA-15** and **-16** (Scheme 14).

### 2.2. Biology

#### 2.2.1. Preliminary Findings [3,6,83]

The biological activity of migrastatin was first reported in 2000 by Imoto et al. [3] These authors showed that migrastatin inhibits the migration of EC17 (mouse esophageal cancer cells), with an IC_50_ of 6 µM in a wound-healing assay (WHA) and of 2 µM in a chemotaxicell chamber assay. However, the same authors reported [83] that a migrastatin sample was contaminated with teleocidin-related impurities, which are known for their anti-migratory activity. After careful purification, they showed an inhibition of cell migration in EC17 cells, pretreated with pure migrastatin for 24 h, with an IC_50_ of 20.5 µM (WHA). In addition, they showed that migrastatin inhibits the growth of EC17 cells, with an IC_50_ of 167.5 µM. These results indicate that inhibition of cell migration was not due to cytotoxicity [83]. In 2006, the same authors reported that migrastatin inhibits the function of P-glycoprotein and is therefore capable of suppressing multidrug resistance (MDR) [6]. They demonstrated that migrastatin increases the intracellular concentration of anticancer drugs vinblastine, vincristine and taxol in P-glycoprotein-overexpressing VJ-300 (vincristine-resistant human epidermoid carcinoma) [84] and P388/VCR (vincristine-resistant mouse leukemia) cells [85]. The cytotoxicity of vincristine and taxol in VJ-300 cells treated with migrastatin (61 µM) increased 40- and 53-fold respectively (migrastatin not toxic up to 102 µM).

#### 2.2.2. Danishefsky’s Work [9,86,87,88]

**Figure 4:**
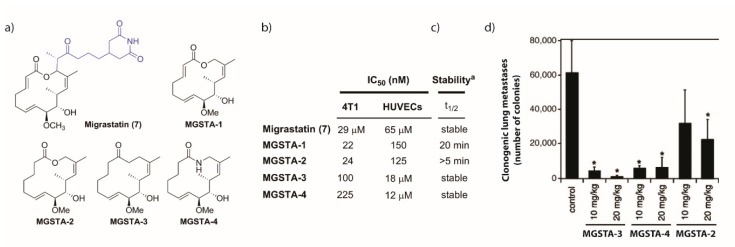
(**a**) Structures of migrastatin (**7**) and migrastatin analogs **MGSTA-1** to **4**; (**b**) Chamber cell migration assay with 4T1 mammary mouse tumor cells and HUVECs; (**c**) metabolic stability of migrastatin and analogs; (**d**) inhibition of breast tumor metastasis (4T1) by **MGSTA-2**, **MGSTA-3**, **MGSTA-4** in a mouse model. Lung metastasis was measured by the 6-thioguanine clonogenic assay. Results are mean ± SD (*n* = 5). *, *p* < 0.01. Adapted with permission from *PNAS*, **2005**, *102*, 3772. Copyright (2005) National Academy of Sciences, U.S.A. ^a^ Intensity of HPLC signal unchanged over 60 min of incubation.

An important breakthrough with respect to the biological activity of migrastatin was made by Danishefsky et al. in 2005 [9]. Through a diverted total synthesis approach (Figure 2), a series of truncated analogs of migrastatin were prepared and tested as inhibitors of cell migration in 4T1 cells (mammary mouse cancer) and HUVECs (human healthy endothelial cells). Migrastatin-core analogs **MGSTA-2** to **4** were ≈ 1000 more potent that migrastatin itself (Figure 4b) and were not cytotoxic up to 20 µM. Interestingly, while migrastatin was stable in mouse plasma, macrolactone **MGSTA-1** and **MGSTA-2** were not. On other hand, macroketone **MGSTA-3** and macrolactam **MGSTA-5** displayed higher stability, as revealed by an unchanged HPLC signal over 60 min of incubation (Figure 4c).

**MGSTA-3** and **MGSTA-4** also inhibited the migration of highly invasive and metastatic cancer cell lines, such as MDA-MB-231 (human breast tumor), Lovo (human colon tumor) and PC-3 (human prostate tumor) in WHAs [86]. Importantly, **MGSTA-3** and **MGSTA-4** did not affect the migration of normal human mammary-gland epithelial cells (MCF-10A), mouse embryonic fibroblasts, or primary mouse leukocytes in WHAs [86].

For in vivo studies [86], the 4T1 mouse mammary model was chosen. In this model, the tumor closely mimics human breast cancer with respect to immunogenicity, metastasis, anatomy and growth characteristics [89]. 4T1 tumors spontaneously metastasize to the lung, bone, brain and liver [90]. **MGSTA-3** and **MGSTA-4** were administrated daily for 20 days at 10 mg/kg or 20 mg/kg. **MGSTA-3** and **MGSTA-4** reduced the metastasized 4T1 cells in the lungs by 91%–99% (measured by 6-thioguanine clonogenic assay) (Figure 4d). Tumor growth was not affected by **MGSTA-3** or **MGSTA-4**, and no obvious side effects were observed. **MGSTA-2**, which was not stable in plasma (Figure 4c), was considerably less effective at inhibiting metastasis (Figure 4). In addition, the authors showed that **MGSTA-3** and **MGSTA-4** block the activation of RAC (Ras-related C3 botolinum toxic substrate 1), a protein involved in lamellipodia formation and therefore in cell migration.

**Figure 5:**
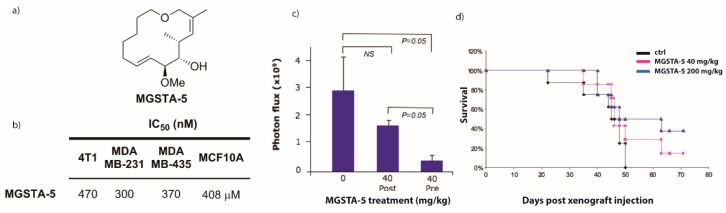
(**a**) Structure of **MGSTA-5**; (**b**) Chamber cell migration assay; (**c**) **MGSTA-5** treatment (40 mg/kg) begun at day 1 (Pre) or day 15 (Post) after tumor inoculation (MDA-MB-231). Primary tumor was resected at 2 weeks and tumor metastasis was determined by bioimaging at 3 weeks; (**d**) **MGSTA-5** treatment (40 or 200 mg/kg) begun at day 1 after tumor inoculation (MDA-MB-231). Primary tumor was resected at 3 weeks and tumor metastasis was determined by bioimaging at 4 weeks. Adapted with permission from *J. Am. Chem. Soc*. **2010**, *132*, 3224. Copyright (2010) American Chemical Society.

A few years later [87], Danishefsky and co-workers prepared the simplified macroether **MGSTA-5** starting from advanced intermediate **8** (Figure 5a). **MGSTA-5** was tested in transwell cell-migration assays against 4T1, MDA-MB-231 (human breast cancer), MDA-MB-435 (human breast cancer) cell lines. **MGSTA-5** inhibited the migration of tested cell lines, with an IC_50_ in the nanomolar range (Figure 5b). Interestingly, a higher concentration of **MGSTA-5** was necessary to inhibit the migration of MCF10A (non-tumor human breast cells), thereby suggesting a specific mechanism of action for transformed cells. **MGSTA-5** also inhibited the proliferation of MDA-MB-231 cells, with an IC_50_ > 100. This observation suggesting that the inhibition of migration in MDA-MB-231 cells is not related to cytotoxicity. **MGSTA-5** inhibited the migration of the highly metastatic breast cancer cell line LM2-4175 (LM2), with an IC_50_ (extrapolated from graph) [87] of around 1.5–2 µM. LM2 cells are aggressive and highly metastatic and are derived from lung metastasis of MDA-MB-231 [91]. The ability of **MGSTA-5** to inhibit tumor metastasis in vivo was assessed using luciferase-based noninvasive whole animal bioluminescent imaging in a xenograft breast cancer model in NOD/SCID mice transplanted with MDA-MB-231 cells stably expressing the HSVTK-eGFP-luciferase (TGL) reporter protein [92]. After inoculation of MDA-MB-231 cells, a group of five mice were treated with **MGSTA-5** (three times/week; 40 mg/kg) from day 1 (pre-treatment). Another group of mice was treated starting from day 15 (post-treatment). At the time of surgical resection, 50% of control mice had metastasis and 85% had tumors invading the muscle layer and peritoneal membrane. The group of mice treated with **MGSTA-5** from day 1 (pre-treatment) showed an 87% reduction in metastases, while that treated with **MGSTA-5** from day 15 (post-treatment) showed a 47% reduction (Figure 5c). Pre-treatment with **MGSTA-5** also had an important effect on overall survival. Mice were pre-treated with **MGSTA-5** at 40 mg/kg and 200 mg/kg. After 50 days, all the control mice had died; however, the overall survival of the groups treated with 40 and 200 mg/kg was 30% and 50%, respectively (Figure 5d). After 9 weeks, a metastatic tumor was detectable in the former group but not in the latter. Importantly, treatment with **MGSTA-5** did not attenuate the growth of the primary tumor [87].

**Figure 6:**
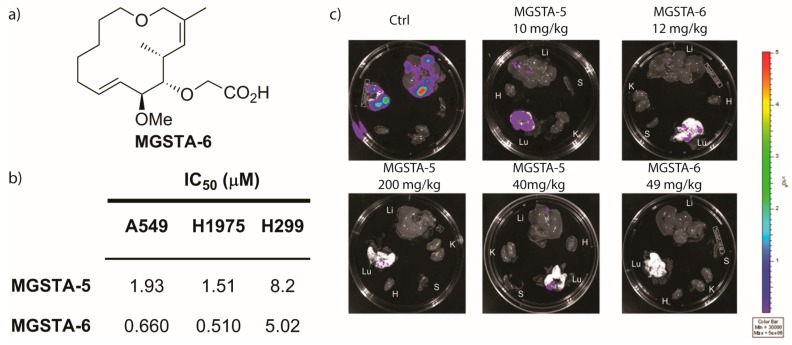
(**a**) Structure of **MGSTA-6** a; (**b**) Chamber cell migration assay with non-small lung carcinoma (NSLC) A549, H1975, H1299 cells; (**c**) Bioluminescent imaging of tumor metastasis at endpoint. Lung (Lu), liver (Li), heart (H), kidneys (K), and spleen (S). Adapted with permission from *PNAS*, **2011**, *108*, 15074.

With the aim to identify new compounds with enhanced solubility, bioavailability and pharmacostability, Danishefsky and co-workers [88] synthesized the carboxymethyl migrastatin-core analog **MGSTA-6** (Figure 6a). The ability of this compound to inhibit cell migration was assessed using the WHA and transwell migration assay in human non-small cell lung carcinoma cells (NSCLC) and compared with that of **MGSTA-5**. Both **MGSTA-5** and **MGSTA-6** efficiently reduced the migration of A549, H1975 and H1299299 cancer cells, with an IC_50_ in the micro and nanomolar range (Figure 6b).

The capacity of **MGSTA-5** and **6** to inhibit tumor metastasis generated from human primary small lung carcinoma cells (SLCL) was also evaluated. Primary tumors were obtained from patients, and cells were stably transduced with a triplefusion protein reporter construct (AC3-TGL) and then transplanted by subcutaneous injection with Matrigel into NOD/SCID IL2R gamma null (NSG) mice. Mice were treated with both **MGSTA-5** and **6** from day 1 at doses of 10, 40 and 200 mg/kg (**MGSTA-5**) and 12 and 49 mg/kg (**MGSTA-6**) (both analogs were administered by intraperitoneal injection three times/week; doses adjusted to molecular weight). Mice were treated for 55 days and monitored by serial noninvasive bioluminescent imaging (BLI). At day 55, mice were killed, and the spread of metastasis in lungs, liver, heart, kidneys and spleen was evaluated using BLI (Figure 6c). Neither analog affected the inhibition of tumor growth. No toxicity was detected in mice treated with **MGSTA-6**. In addition, treatment with the low dose of **MGSTA-6** (12 mg/kg) was approximatively four times more potent than **MGSTA-5** at the same dose (10 mg/kg).

#### 2.2.3. Mechanism of Action [93]

**Figure 7:**
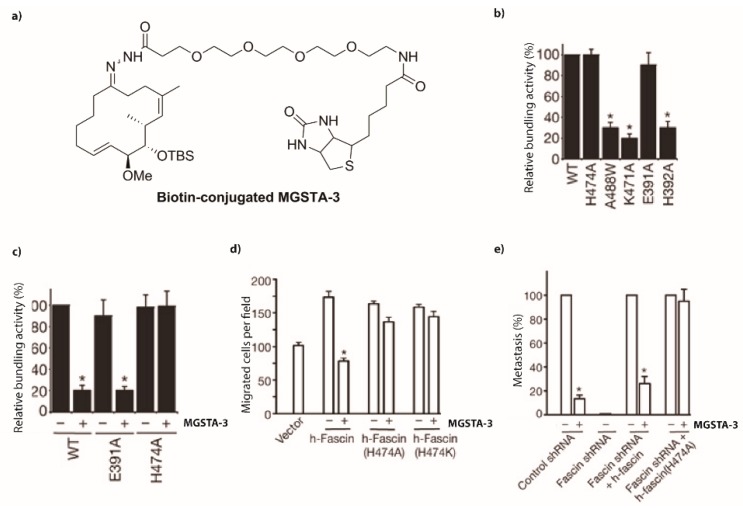
(**a**) Structure of Biotin-conjugated **MGSTA-3**; (**b**) Quantification of actin bundling assay for the wild-type fascin and mutants; results are means and ± SD (*n* = 3); * *p* < 0.05 (**c**) Mutant sensitivity to **MGSTA-3**. Wild-type fascin and the E391A and H474A mutants of fascin were assayed for their actin-bundling activity in the absence or presence of **MGSTA-3** (10 µM); results are means and ±SD (*n* = 3). * *p* < 0.05; (**d**) Boyden chamber cell migration assay of mouse fascin shRNA-treated 4T1 cells transfected with various mutants of GFP–human fascin (h-fascin) in the presence or absence of **MGSTA-3** (10 µM); results are means and ± SD (*n* = 5) *p* < 0.05; (**e**) Tumor metastasis assay with mouse fascin shRNA-treated 4T1 cells overexpressing wild-type human fascin or fascin (H474A) mutant in the presence or absence of **MGSTA-3** (10 mg/kg). Comparison of the fascin shRNA group with the control shRNA group. Results are means and ±SD (*n* = 5~6). *, *p* < 0.05 Adapted by permission from Macmillan Publishers Ltd: *Nature*, **2010**, *464*, 1062, copyright (2010) http://www.nature.com/.

In 2010, Chen and co-worker [93] attempted to elucidate the mechanism of action of **MGSTA-3**. The authors claimed that the target of **MGSTA-3** was fascin. Fascin is an actin-bundling protein responsible for cell migration and therefore for cell invasion and metastasis. Fascin mRNA transcript and protein levels are elevated in aggressive tumors [94,95], and overexpression of fascin is also linked to increased cell migration and invasion [96,97]. Using an affinity protein purification approach, they demonstrated that a **MGSTA-3** biotin-labeled analog [9] (Figure 7a) binds to fascin in cancer cell extracts. F-actin pelleting assays [98] revealed that **MGSTA-3** significantly decreases fascin-induced bundling of F-actin polymers. The authors also published an X-ray crystal structure of fascin co-crystalized with **MGSTA-3** and claimed that **MGSTA-3** binds at the same site of actin. However, this X-ray structure was found to be incorrect and was therefore retracted [93], since the chemical structure of **MGSTA-3** bound to fascin shown in the X-ray picture was not **MGSTA-3** but rather its isomer [99]. Furthermore, the authors showed that selective mutation of fascin in the proposed region of binding reduced the actin-bundling activity of fascin (mutation on H392, Lys471, Ala488, F-actin pelleting assay, Figure 7b). On other hand, mutation of His 474 to Ala did not reduce the activity of fascin but rendered fascin resistant to **MGSTA-3** treatment (Figure 7c). These results were also validated in 4T1 cancer cells in a boyden chamber assay (Figure 7d).

**Figure 8:**
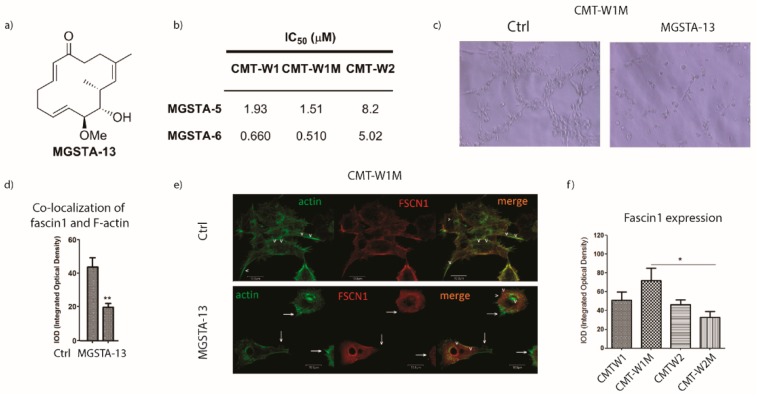
(**a**) Structure of **MGSTA-13**; (**b**) Chamber cell migration assay with canine mammary cancer cell lines; (**c**) Growth characteristics of CMT-W1, cell lines cultured on Matrigel and treated with **MGSTA-13** 100 µM for 24 h; (**d**) Quantification of fascin1 and F-actin co-localization at merge images; the unpaired t-test was applied. *p* < 0.01 (**e**) Representative confocal microscopy images of cytoskeletal protein F-actin and fascin1 in CMT-W1M canine carcinoma cell line; (**f**) Expression of phospho-FSCN1(Ser39) in canine mammary cancer cell lines; the unpaired t-test was applied. * *p* < 0.05. Adapted from *PLoS ONE*, **2013**, *8*, e76789.

Knockdown of fascin using short-hairpin RNAs (shRNA) decreased cell migration. This effect was rescued by transfection of wild-type human fascin cDNA or H477A/H477K human fascin cDNA. Importantly, rescued migration of WT fascin was sensitive to **MGSTA-3** while rescued migration by H477A or H477K fascin was not (Figure 7d). Similar results were obtained in vivo using various fascin mutants in mouse fascin shRNA-treated 4T1 cells in the presence or absence of **MGSTA-3** (Figure 7e). In 2013, Król and co-workers [19] examined the effect of **MGSTA-2** to **3**, **MGSTA-8** to **10** and **MGSTA-13** in canine mammary cell lines. The study was carried out using CMT-W1 and CMT-W2 cells and their corresponding lung metastasis CMT-W1M and CMT-W2M cells [100,101]. A preliminary screen using the wound-healing assay (WHA) showed that **MGSTA-3** and **MGSTA-13** were the most promising analogs. Further single concentration studies (Boyden chamber assay) showed that **MGSTA-13** was the most promising compound. **MGSTA-13** inhibited cell migration of CMT-W1, CMT-W1M, CMT-W2 cells, with an IC_50_ in the molar range (Figure 8b). Surprisingly, no effect was observed in CMT-W2M cells. They next examined the invasive phenotype [102,103] of CMT-W1, CMT-W1M, CMT-W2 cells cultured on reconstituted basement membrane (Matrigel^TM^) in the presence or the absence of **MGSTA-13**. After 24 h, cells cultured in control conditions showed typical branching formation. In contrast, all cells treated with **MGSTA-13** showed a remarkable inhibition of branch formation (Figure 7c). This observation indicates that this compound has an effect on actin machinery, which is responsible for filopodia formation and thus for cell migration. Given these findings, the authors focused their attention on fascin, an actin-bundling protein responsible for the development and maintenance of straight and tight F-actin bundles [104,105,106]. Using confocal microscopy, they demonstrated that actin strongly co-localized with fascin1 in CMTW1 cells, while after treatment with **MGSTA-3** this co-localization decreased dramatically (Figure 8d). In addition, CMT-W1 cells in control conditions showed several filopodia and protrusions, which disappeared after treatment with **MGSTA-13** (Figure 8e). Similar results were obtained with CMT-W1 and CMT-W2 cells. With these results in hand, the authors analyzed the expression of phospho-fascin1 protein (phospho-FSCN1(ser39)) in the four cell lines. The expression of fascin1 in CMT-W2M cells, which were not sensitive to **MGSTA-3** and **13**, was lower than in the other cell lines (Figure 8f).

In 2014, Murphy and Anderson [14] studied the effect of **MGSTA-3** on epithelial cadherin (E-cadherin) dynamics in vivo and in vitro. Misregulation of epithelial E-cadherin is associated with the ability of cancer cells to detach from the primary tumor and to become invasive and metastatic [107,108,109]. E-Cadherin is a cell-cell adhesion protein involved in the maintenance of the epithelial architecture [110]. Reduced levels of E-cadherin have been found in colon, breast, prostate and ovarian cancer [111,112,113,114]. Pancreatic ductal adenocarcinoma (PDAC) cells were treated with **MGSTA-3**, and E-cadherin dynamics was studied using fluorescence recovery after bleaching (FRAP). These cells are highly invasive because of expression of a mutant form of the tumor suppressor p53 [115]. **MGSTA-3** had no effect on the immobile fraction of E-cadherin, a measure of the amount of E-cadherin immobilized at cell-cell junctions. PDAC cells were therefore injected subcutaneously into nude mice, and tumors were allowed to grow for seven days. The mice were treated with **MGSTA-3** (20 mg/kg) for three days, and E-cadherin dynamics were studied using FRAP. **MGSTA-3** treatment increased the immobile fraction, an effect expected to strengthen cell-cell adhesion and therefore impede metastasis.

**Figure 9:**
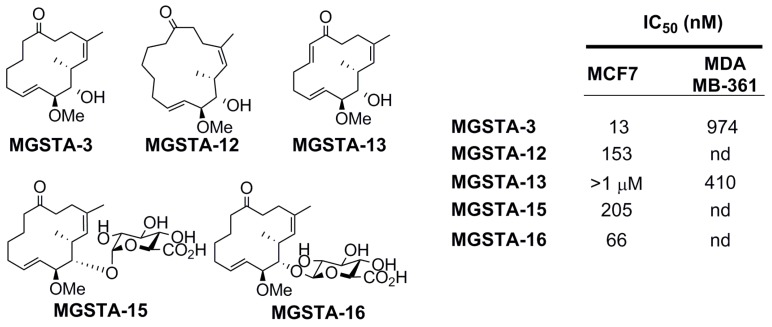
Structures of selected migrastatin analogs and effect on human breast cancer cell lines MCF7 and MDA MB-361 assessed with Boyden chamber assay.

In 2015, Murphy and co-workers reported the biological activity of several migrastatin core analogs. **MGSTA-8** to **16** were tested against three breast (MCF7, MCF7-Dox, MDA-MB361) and one pancreatic (HPAC) cancer cell lines in WHA. All analogs inhibited cell migration, and none were toxic up to 100 µM. Selected analogs (**MGSTA-3**, **MGSTA-12** to **13**, **MGSTA-15** to **16**) were therefore tested in transwell assays in MCF7, MDA-MB-361 and HPCA cells (Figure 9). All these compounds inhibited cell migration in the nanomolar range, with the exception of **MGSTA-13** in MCF-7 cells. Since **MSTA-13** inhibited cell migration in the highly metastatic MDA-MB-361 cell but not in the less invasive MCF7 cells, the authors claimed that this effect could be related to cytoskeleton proteins targeted by migrastatin analogs [14,93]. Interestingly, **MGSTA-13** was tested against a panel of 55 targets known to be related to adverse drug reactions (ADRs). **MGSTA-13** showed only weak inhibitory capacity over adenosine receptor A2A (27%) and prostanoid EP4 receptor (39%). It has been reported that a promiscuity index (percentage of targets giving more than 50% inhibition at 10 µM in a set of at least 50 targets) of more than 20% is linked to market withdrawal and clinical trial failure. Unsaturated macroketone **MGSTA-13** registered a promiscuity index of between 0% and 5%, and is therefore a promising candidate for further biological studies.

The biological activity of migrastatin-core analogs has attracted the attention of cancer biologists in recent years. Several synthetic approaches have been developed for these compounds, and their relatively simple structure has allowed the preparation of small libraries and scale-up for in vivo studies.

Migrastatin analogs act as anti-metastatic agents by inhibition of the cell motility machinery and they have no effect in tumor growth or proliferation. The mechanism by which migrastatin analogs interfere with cell migration is still debated and further studies are necessary in order to confirm their primary target. Preliminary data suggest that migrastatin analogs selectively reduced cell migration in cancer cells without interfering with non-transformed cells. New findings in this direction will definitely help medicinal chemist to design new analogs with enhanced biological activity and stability.

In addition, this class of compound displayed low cytotoxic activity and recently it have been demonstrated that **MGSTA-13** present low affinity for several targets related to adverse drug reactions (ADRs). Since high affinity of targets related with ADRs result in preclinical/clinical or market withdrawal, migrastatin analogs could be valuable molecules for further preclinical development. Efforts in this direction could lead to the identification of preclinical candidates with enhanced safety profile.

We also believe that the preparation of synthetic migrastatin analogs with enhanced water solubility and plasma stability it is also necessary. It has been demonstrated that free hydroxyl group in migrastatin core could be modified with the introduction of a carboxymethyl moiety or sugar moiety without drastically affect the biological activity. Moreover, modification of hydrophobic left hand side of migrastin-core analogs has not be explored.

From the clinical point of view, migrastatin analogs could act as prophylactic agents that prevent tumor cells to metastasize. Safe and efficient migrastatin analogs could be administrated after liver or lung metastasis resection and this could prevent metastasis relapse. Alternatively, migrastatin analogs could be administrated during or after chemotherapy standard treatment.

We hope that this review serves to inspire the preparation of novel analogs with enhanced pharmacological properties, thus paving the way for their application in the treatment of patients with metastatic cancer.

## Figures and Tables

**Table 1:** Synthetic key aspects for the preparation of advanced precursor **8**.

Year	Authors	n^0^ Steps	Overal Yield	Key Steps	Notes	Reference
2004	Danishefsky et al.	10	22%	LACDAC reaction, Ferrier rearrangement	Multi-gram scale synthesis	[8,9,16]
2006	Cossy et al.	11 (+4) ^a^	11%	Stereoselective crotylmetalation, RCM	-	[10]
2007	Cossy et al.	11 (+4) ^a^	35%	Stereoselective crotylmetalation, Still-Gennari olefination	Entry to isomigrastatin analogues, gram scale	[17]
2006	Lqbal et al.	12	8%	Evans aldol condensation and distereoselective vinylmagnesium bromide additon	Entry to isomigrastatin analogues	[11]
2010	Dias et al.	14	1.2%	Upjohn dihydroxilation, Horner-Wadsworth-Emmons olefination	Entry to isomigrastatin-core epimers, gram scale	[12]
2013	Lqbal et al.	11	5.9%	Pd(II) catalyzed intramolecular C-H oxidation	Entry to migrastatin-core with different functional groups	[13]
2014	Murphy et al.	9	30%	Brown alcoxyallylation, HWE, Zinc catalyzed asimmetric desymmetrization	Entry to isomigrastatin analogues, gram scale	[14,18,19]

^a^ Precursor of advanced intermediate **8**.

## Abbreviations

**List of Abbreviations Used**

2,6-lutidine	2,6-dimethylpyridine
BAIB	(Diacetoxyiodo) benzene
CSA	camphorsulfuric acid
DCC	*N*,*N*’-dicyclohexylcarbodiimide
DIBAL	diisobutylaluminium hydride
DIPEA	*N*,*N*-Diisopropylethylamine
DMP	Dess-Martin periodinane
DMAP	4-dimethylaminopyridine
DPPA	diphenylphosphoryl azide
DTBMP	2,6-di-*tert*-butyl-4-methylpyridine
DBU	1,8-Diazabicyclo[5.4.0]undec-7-ene
EDC	1-Ethyl-3-(3-dimethylaminopropyl)carbodiimide
Grubbs II	(1,3-Bis(2,4,6-trimethylphenyl)-2-imidazolidinylidene)dichloro(phenylmethylene) (tricyclohexylphosphine) ruthenium
(+)Ipc_2_BOMe	(+)-*B*-methoxydiisopinocampheylborane
MS	molecular sieves
MeOTf	methyl trifluormethanesulfonate
MOMCl	chloromethyl methyl ether
NMO	*N*-methylmorpholine-*N*-Oxide
Proton sponge	1,8-bis(dimethylamino)naphthalene
TBDPSCl	*tert*-butyldiphenylsilyl chloride
TBAF	tetrabutylammonium fluoride
TBSCl	*tert*-butyldimethylsilyl chloride
TBSOTf	*tert*-butyldimethylsilyl trifluoromethanesulfonate
Tebbe reagent	Bis(cyclopentadienyl)-μ-chloro-(dimethylaluminum)-μ-methylenetitanium
TEMPO	2,2,6,6,-tetramethyl-1-piperidinyloxy
TPAP	tetrapropylammonium perruthenate
*p*-TSA	*p*-toluenesulfonic acid
White catalyst	1,2-bis(phenylsulfinyl)ethane palladium (II) acetate
WHA	wound-healing assay

**List of Cell Lines**

4T1	(mammary mouse cancer)
A549	(lung carcinoma)
CMT-W1	(canine mammary cancer)
CMT-W2	(canine mammary cancer)
CMT-W1M	(canine lung metastasis)
CMT-W2M	(canine lung metastasis)
EC17	(mouse esophageal cancer)
H1299	(lung cancer)
H1975	(lung adenocarcinoma)
HPAC	(human pancreas adenocarcinoma)
HUVECs	(human healthy endothelial cells)
LM2-4175	(lung metastatic cells derived from MDA-MB-231)
Lovo	(human colon cancer)
MCF7	(human breast cancer)
MDAB-MB-361	(human breast cancer)
MCF-10A normal human mammary-gland epithelial	
MDA-MB-231	(human breast cancer)
MDA-MB-435	(human breast cancer)
P388/VCR	(vincristine-resistant mouse leukemia)
PC-3	(human prostate cancer)
PDAC	(pancreatic ductal adenocarcinoma)
VJ-300	(vincristine-resistant human epidermoid carcinoma)
